# Prevalence, Risk Factors, and Antibiogram Analysis of Bovine Mastitis in Northern Bangladesh

**DOI:** 10.3390/vetsci12121201

**Published:** 2025-12-15

**Authors:** Md. Ashraf Zaman Faruk, Md. Mizanur Rahman Manu, Farzana Afroz, Md. Wajed Ali, Md Atiqul Haque, Danishuddin, Md Azizul Haque

**Affiliations:** 1 Department of Livestock Services, Upazila Livestock Office and Veterinary Hospital, Naldanga 6403, Bangladesh; faruk.unibd@yahoo.com; 2 Department of Microbiology, Hajee Mohammad Danesh Science and Technology University, Dinajpur 5200, Bangladesh; manuhstu92@gmail.com (M.M.R.M.); farzana.afroz2010@gmail.com (F.A.); atique@hstu.ac.bd (M.A.H.); 3 Poultry Research Centre, Bangladesh Livestock Research Institute (BLRI), Dhaka 1341, Bangladesh; 4 Department of Livestock Services, Veterinary Training Institute, Alamdanga 7210, Bangladesh; wajedrubd@gmail.com; 5 Department of Biotechnology, Yeungnam University, Gyeongsan 38544, Republic of Korea

**Keywords:** antibiogram study, California Mastitis Test, clinical mastitis, subclinical mastitis

## Abstract

Mastitis remains a major constraint to dairy production in Bangladesh, particularly in high-demand regions. This study investigated the prevalence, risk factors, and antimicrobial resistance patterns of major pathogens associated with bovine mastitis in Northern Bangladesh. A total of 488 milk samples from 122 randomly selected milking cows were tested through clinical inspection and the California Mastitis Test (CMT). Positive samples were subjected to bacterial culture and antimicrobial susceptibility testing using the disc diffusion method. The overall cow-level prevalence was 35.24%, with 27.86% sub-clinical and 7.37% clinical cases. At the quarter level, prevalence was 21.72%. Among 166 bacterial isolates, *Staphylococcus* spp. (41.56%) was predominant, followed by *Streptococcus* spp. (23.49%), *E. coli* (17.47%), *Klebsiella* spp. (12.65%) and *Bacillus* spp. (4.82%). Gentamicin (100%) and Streptomycin (92.20%) were the most effective antibiotics, whereas Cefixime (100%) and Amoxicillin (87.40%) showed the highest resistance rates. Major risk factors included late lactation stage, high milk yield, previous mastitis history, and poor hygiene. In contrast, breed, age, and parity were not significant. These findings highlight a high prevalence of mastitis, predominance of *Staphylococcus* spp., and rising antimicrobial resistance, identify associated risk factors, and rising antimicrobial resistance profiles in Northern Bangladesh.

## Simple Summary

Mastitis is a serious threat to the dairy industry in Bangladesh, where milk production remains insufficient to meet the growing demand. Although several studies have addressed bovine mastitis across the country, limited information is available from the northern region. This study investigated the prevalence, risk factors, and antibiotic resistance patterns of mastitis-causing bacteria in this area. Among 122 cows tested, about one-third were affected, mostly with subclinical infections. The major pathogens identified were *Staphylococcus* and *Streptococcus* spp. Antibiotic sensitivity testing showed that Gentamicin and Streptomycin were effective, while high resistance was observed against Amoxicillin, Penicillin, and Cefixime. Cows in late lactation, producing high milk yields, or with a history of mastitis were more prone to infection. Poor hygiene, unclean floors, and improper milking practices significantly increased the risk. The study highlights the urgent need for improved farm hygiene, regular mastitis screening, and responsible antibiotic use to reduce infection rates and ensure sustainable dairy production in northern Bangladesh.

## 1. Introduction

Bangladesh has an impressive livestock density, with 25.7 million cattle totaling of which 3.53 million are mostly milking cows [1]. As per data from the Department of Livestock Services (DLS), Bangladesh produced 15.38 million metric tons of milk in the 2024–2025 fiscal year. Milk production has increased almost threefold between 2015 and 2025, with a cumulative average growth rate of 7.81 percent [2]. Despite expanded production, the industry still needs to meet domestic demand. Presently, the availability of milk per person is 239.29 mL/day, below the recommended minimum daily intake of 250 mL. In contrast to the current supply of 155.38 lakh metric tons (1 lakh = 100,000), the market demand for milk is 162.33 lakh metric tons [2]. This deficit can be attributed to several factors, including a lack of land, limited access to feeds and medications, poor product prices, unsuitable cattle breeds for high milk output, insufficient investment, and a high prevalence of diseases [3]. Mastitis, particularly in developing nations like Bangladesh, is a highly prevalent multi-etiological and complicated disease in the dairy industry [4,5]. Reduced milk production, poor quality milk, increased treatment and veterinary services costs, increased unexpected culling rate, increased risk of antimicrobial resistance, occasional deaths, and compromised animal welfare are all consequences that lead to the most costly and devastating diseases [6,7]. For example, in Egypt, milk yield losses from subclinical mastitis were significant, reaching over 20 million LE (Egyptian pound) annually. In the US, total economic costs in the first 30 days of lactation were calculated at USD 444 per cow, with indirect costs such as premature culling and milk production loss being major contributors [8,9]. According to a report by the University of Glasgow, mastitis is estimated to impose an economic burden of USD 19.7 to USD 32 billion per year on the global dairy industry [10]. Meanwhile, projected yearly economic losses in Bangladesh from mastitis are BDT 122.6 (USD 2.11) million [5].

Mastitis, also known as parenchymal inflammation of mammalian glands, is typically accompanied by pathological changes in the glandular tissue of the udder as well as changes in the physical, chemical, and bacteriological characteristics of milk [11]. The condition is frequently linked to management practices, pathogens, hosts, and other factors such as genetics, teat or udder form, and environment [12,13]. The most frequently isolated mastitis-causing bacterial pathogens are *Staphylococcus aureus*, *Streptococcus agalactiae*, *Streptococcus dysgalactiae*, *Mycoplasma* spp., *Corynebacterium bovis*, *Escherichia coli*, *Klebsiella* spp., *Proteus* spp., and *Enterobacter* spp. [14,15].

Previously, many studies have been carried out to investigate the frequency and associated risk factors of mastitis in developing countries like India [16], Pakistan [15,17], Sri Lanka [18,19], Nepal [20], Kenya [21], Brazil [22], and Ethiopia [14]. Although there have been many investigations into bovine mastitis in Bangladesh, these have been speculative only on the prevalence and risk factors or even bacteriological examinations [4,7,23], with few potential studies on antibiogram profiles reported [24]. Antibiotics are widely used for the treatment of diseases, including mastitis, as preventive measures, and as growth promotion [25]. Multidrug-resistant bacteria, on the other hand, can emerge as a global threat due to the overuse and inappropriate use of antibiotics [12,26]. Consequently, antibiogram profiles against mastitis-causing pathogens have been reported worldwide [15,17,27].

Most studies to date have focused on central or established dairy regions in Bangladesh, without adequately covering other newly growing dairy hubs. Furthermore, there is no published data on the prevalence, severity, or distribution of mastitis in the Northern region of Bangladesh, especially in the Dinajpur district and its surrounding areas [4,5,7,23,24,28]. However, Bangladesh has no national surveillance program for bovine mastitis prevalence nor national guidelines for using antibiotics to treat mastitis. This study addresses this gap by examining the prevalence of both clinical and subclinical mastitis in Northern Bangladesh, along with identifying the associated risk factors, main causative pathogens, and antibiogram profiles.

## 2. Materials and Methods

### 2.1. Ethics Statement

The experimental protocols for this research were reviewed and approved by the Ethical Reviewing Board of the Institutional Animal Care and Use Committee (IACUC) at Hajee Mohammad Danesh Science and Technology University. The approval was granted under the code HSTU/IRT/3630, and all procedures were conducted in accordance with the ethical guidelines and animal welfare protocols to minimize distress and ensure the humane treatment of the animals.

### 2.2. Study Area and Sampling Details

**Figure 1:**
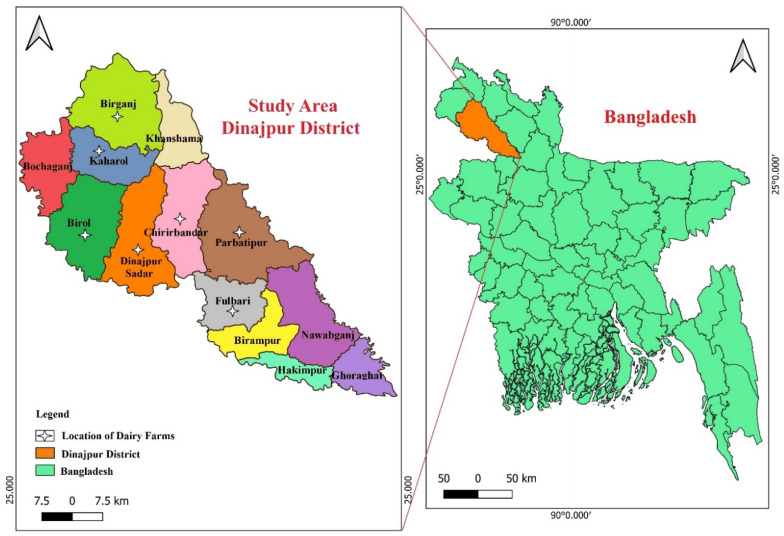
Map of the study area.

This study was undertaken in the Dinajpur district (25.6279° N and 88.6332° E) of the Rangpur division, about 340 km from Dhaka, the capital of Bangladesh [29], which experiences a subtropical climate with distinct wet (June–September) and dry (October–March) seasons, marked by hot, humid monsoons and mild, clear winters. Average temperatures range from 50 °F to 97 °F, with most of the annual ~2340 mm rainfall occurring during the monsoon. The present study was conducted over a full seasonal cycle. A total of 122 lactating cows, including Holstein Friesian cross (n = 73), Sahiwal cross (n = 16), Jersey cross (n = 18), and local breed (n = 15), were randomly selected from the 43 dairy farms in Dinajpur town and six surrounding Upazilas (Figure 1). Farms were randomly selected from areas in Northern Bangladesh where new milk hubs have been established over the years, and those actively operating and regularly milking cows. The number of cows sampled from each farm was determined using a proportionate random sampling method, where the sampling fraction for each farm was based on the total number of lactating cows present during the visit, ensuring equal probability of inclusion. Only active dairy farms operating under newly established milk hubs were selected. For every enrolled cow, breed, age, parity, stage of lactation, milk production capacity, and previous history of mastitis were recorded, and both clinically healthy and mastitic cows were included to ensure a comprehensive assessment of the population. Initially, the CMT test was performed to classify individual quarter samples as subclinical or clinical mastitis cases. Following CMT classification, milk samples were aseptically collected using standardized procedures [30,31]. Briefly, after sanitizing the udder and teat with 70% ethyl alcohol and stripping the first 2–3 streams of milk, a teatful of milk (5–10 mL) was milked by hand into sterile wide-mouth vials from each quarter and marked accordingly [32]. Immediately, milk specimens were chilled in ice boxes with cold packs, stored at 4 °C, and brought to the Bacteriology Laboratory, Hajee Mohammad Danesh Science and Technology University, Dinajpur, Bangladesh, for further analysis.

### 2.3. Validated Questionnaires

A cross-sectional study was performed to choose interview respondents, and 43 farms were arbitrarily selected. Data on the farm-related factors, including rearing, housing (floor type, bedding), milking techniques, floor cleaning frequency, udder and hand washing before milking, milking type, teat dipping after milking, dry therapy, farm hygiene, and animal-related factors, including breed, parity, age, milk yield, lactation stage and previous history of mastitis were gathered (Table S1) by registered Veterinarian.

### 2.4. Clinical and Sub-Clinical Mastitis Detection

Direct clinical signs and symptoms of the milk samples (clots, flakes, blood, etc.) allowed for the diagnosis of clinical mastitis (CM) as mentioned earlier by Quinn et al. [13], while sub-clinical mastitis (SCM) was investigated using CMT.

### 2.5. California Mastitis Test (CMT)

The CMT procedure was followed as directed in the manufacturer’s instructions (Leukocyst^®^, Synbiotics Corporation, France). Briefly, equal quantities of CMT reagent and milk (approximately 2 mL from each quarter) were added to four shallow cups of CMT paddles. The paddle was then gently moved in a circular motion on a horizontal plane for a short period (a few seconds) to mix the ingredients. The CMT response occurred immediately and was categorized as negative (0), trace (T), 1+ (weak positive), 2+ (distinct positive), or 3+ (strong positive) for each half gland (teat), based on how much gel was produced and how thick it was [27]. A CMT score of 1+ or higher was counted as positive for SCM; conversely, negative [31]. To minimize variability in CMT readings, all tests were performed by a single trained investigator following standardized procedures.

### 2.6. Bacteriological Examination

The milk samples were examined according to the guidelines of the National Mastitis Council [32]. A loopful (10 µL) of milk was inoculated on the surface of Blood Agar (5% sheep blood) (Oxoid, Hampshire, UK) and incubated at 37 °C for 24 h. The plates were investigated for growth characteristics and colony morphology, including size, shape, pigment production, and hemolysis pattern. To obtain pure colonies, the individual colonies were subcultured on Nutrient Agar (Oxoid, UK) (Figure S1). After 24–48 h of incubation, each culture was subjected to a Gram stain to determine Gram-positive or Gram-negative bacteria (Figure S2). The culture was then grown in selective media such as Mannitol Salt Agar (HiMedia, Thane, India), Edwards medium (HiMedia, India), MacConkey agar (HiMedia, India), Eosin Methylene Blue agar (HiMedia, India), Brilliant Green Agar (HiMedia, India), and the following biochemical tests were performed: Citrate test, Indole test, TSI Agar test, Methyl red, Voges-Proskauer, Catalase test, Lecithinase, and Starch test (Tables S2 and S3 and Figures S3–S13).

### 2.7. Antibiotic Sensitivity Test

The antibiotic sensitivity was measured using the Kirby-Bauer disc diffusion method with Muller-Hinton agar (HiMedia, India). Following overnight incubation at 37 °C, the inhibition zones surrounding each antimicrobial disc were determined in millimeters (mm) and classified as sensitive or resistant following Clinical & Laboratory Standards Institute (CLSI) guidelines [33]. All isolates were examined for sensitivity to ten antibiotics, namely Ampicillin (AMP, 10 µg), Ciprofloxacin (CIP, 5 µg), Amoxycillin (AMX, 30 µg), Gentamycin (GEN, 10 µg), Streptomycin (STM, 10 µg), Cefixime (CFM, 5 µg), Kanamycin (KAN, 30 µg), Penicillin (PCN, 10 µg), Colistin (CST, 10 µg), and Tetracycline (TET, 30 µg) that were frequently used in the investigated area to treat various diseases.

### 2.8. Data Analysis

Data from laboratory analyses and survey responses were initially compiled in Microsoft Excel and then transferred to STATA (Version 13.0, StataCorp, College Station, TX, USA) for detailed statistical analysis. Prevalence rates were computed as percentage values. Associations between response and explanatory variables were assessed using Chi-square tests (χ^2^), and a binary logistic regression model was employed to evaluate the influence of potential risk factors (both in animal and farm related) on mastitis prevalence, where the dependent variable was mastitis status (0 = healthy, 1 = mastitic), and independent variables included breed, age, parity, stage of lactation, milk production capacity, previous history of mastitis. Odds ratios (ORs) were calculated to quantify the strength of the association between specific risk factors and mastitis prevalence, using the lowest prevalence group as the reference category. Statistical significance was established at *p* < 0.05 and *p* < 0.01, with 95% confidence intervals (CIs) reported for all estimates.

A two-step strategy was used for risk-factor analysis. First, univariate logistic regression screened variables with *p* < 0.2, following the standard purposeful-selection approach to retain potentially relevant predictors or confounders. These variables were then included in a multivariate logistic regression model to adjust for confounding, with the final model retaining only those factors significant at *p* < 0.05.

## 3. Results

### 3.1. Prevalence of Mastitis

This investigation examined the prevalence of mastitis in 488 quarters from 122 cows across 43 farms. Based on the CMT and clinical examination, the overall occurrence of mastitis at the cow level was 35.24% (43/122), with SCM and CM accounting for 27.86% (34/122) and 7.37% (9/122), respectively. At the quarter level, the occurrence of mastitis was 21.72% (106/488), with SCM at 17.82% (87/488) and CM at 3.89% (19/488). Table 1 details the prevalence rates of both CM and SCM at the cow and quarter levels.

### 3.2. Risk Factors Associated with the Prevalence of Mastitis

Table 2 and Table 3 display the correlation between animal and farm-related variables and mastitis outbreaks. The study found no statistically significant differences (*p* > 0.05) in mastitis prevalence across breeds and ages. However, among the four breeds, Friesian crosses exhibited the highest prevalence of mastitis (46.57%), and this association was statistically significant (*p* < 0.05) only. In contrast, Jersey crosses (27.77%) and local breeds (13.33%) showed comparatively lower prevalence rates; however, these differences were not statistically significant. The OR of mastitis infection is 6.10 (95% CI: 1.29–28.79) in Friesian crosses, 2.69 (95% CI: 0.44–16.39) in Jersey crosses, and 1.08 (95% CI: 0.13–8.80) in local breeds, with Sahiwal crosses as the reference. Additionally, the likelihood of infection increased with age and parity, with animals over 9 years old (39.13%) and those with ≥7 parities (80.0%) showing higher prevalence rates. Cows older than 9 years have an OR of 1.39 (95% CI; 0.74–2.63) for mastitis infection compared to 6–9 years old cows, which have an OR of 1.22 (95% CI; 0.70–2.12), using cows aged 2–5 years as the baseline. The risk of mastitis infection was significantly (*p* < 0.05) higher in cows with a parity of seven or more (OR: 10.73, 95% CI: 4.47–25.59) and in those with a parity between four to six (OR: 2.40, 95% CI: 1.18–4.85) when compared to cows with a parity of 1–3.

Mastitis incidence was significantly correlated (*p* < 0.01) with lactation stage, milk yield, and previous disease history (Table 2). Mastitis occurred more frequently in early (47.72%) and late (55.55%) lactation cows compared to mid-lactation cows (13.72%). Importantly, cows producing more than 10 L of milk (62.16%) were more prone to mastitis than those producing less milk (25.42% for 5–10 L and 19.23% for <5 L). In cows producing more than 10 L of milk, the odds of mastitis are 6.90 times higher (95% CI: 3.46–12.78, *p* = 0.0008) compared to those producing between 5–10 L or less than 5 L. Cows with a previous history of mastitis were about 61.53% more likely to have mastitis than those without (28.12%). Mastitis was 4.10 times more common (95% CI: 1.76–9.53, *p* = 0.001) in cows with a previous CM or SCM history of the disease than in non-stricken cows.

Regarding farm-related factors, husbandry practices, bedding, teat dipping, and dry cow therapy were not statistically significant (*p* > 0.05), while floor type, cleaning frequency of the floor, udder washing, hand washing, milking type, and farm hygiene were significantly correlated (*p* < 0.01) with mastitis occurrence (Table 3). Poor floor type, washing systems, and farm hygiene practices contributed to over 70% of mastitis cases. Cows with irregular udder washing (79.48%), weekly floor cleaning (88.0%), and only partial milking (66.66%) had higher mastitis prevalence compared to those with regular udder cleaning (14.45%), floor cleaning (21.65%), and complete milking (20.48%). The OR indicated that a concrete floor was 10.09 times (95% CI: 3.07–33.07, *p* = 0.00003) more likely to cause mastitis in dairy cows than muddy floor types. The OR of 26.54 indicates that cows on farms where the floor was cleaned weekly had approximately 26.54 times higher odds of developing mastitis compared with farms that cleaned the floor daily (95% CI: 7.24–97.33, *p* = 0.0001). Owners relying solely on washing to maintain udder cleanliness had 17.10 times (95% CI: 3.47–25.1, *p* = 0.0005) higher odds of mastitis prevalence. Complete milking and good farm hygiene practices decreased the risk of mastitis in cows by 7.76 times (95% CI: 6.73–36.85, *p* = 0.002) and 9.57 times (95% CI: 3.91–23.36, *p* = 0.001) compared to partial milking and poor hygiene.

### 3.3. Prevalence of Causative Bacteria of Mastitis

**Figure 2:**
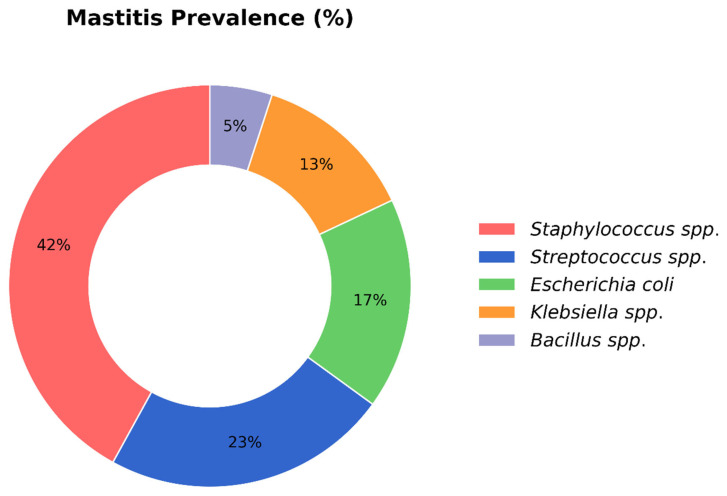
The percentage of bacterial isolates causing bovine mastitis.

**Figure 3:**
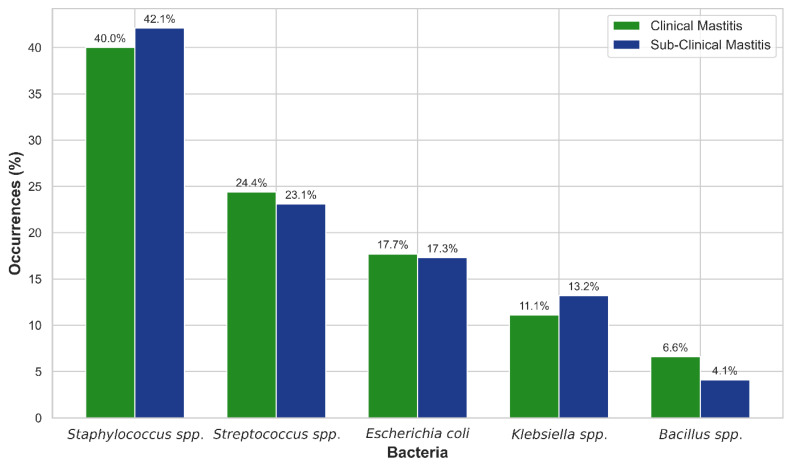
Distribution of bacterial isolates responsible for clinical and subclinical mastitis. The number at the top of each bar represents the positive rate for the corresponding bacteria.

Out of 166 isolates, the bacterial strains identified were *Staphylococcus* spp. (42%), *Streptococcus* spp. (23%), *Escherichia coli* (17%), *Klebsiella* spp. (13%), and *Bacillus* spp. (5%) (Figure 2 and Table S4). *Staphylococcus* spp. was the most predominant bacterium in both CM and SCM cases, at 40% and 42.1%, respectively, followed by *Streptococcus* spp. (23.1% and 24.4%), *E. coli* (17.3% and 17.7%), *Klebsiella* spp. (11.1% and 13.2%), and *Bacillus* spp. (4.1% and 6.6%) (Figure 3 and Table S4).

### 3.4. Antibiogram Profile of Isolated Bacteria

**Figure 4:**
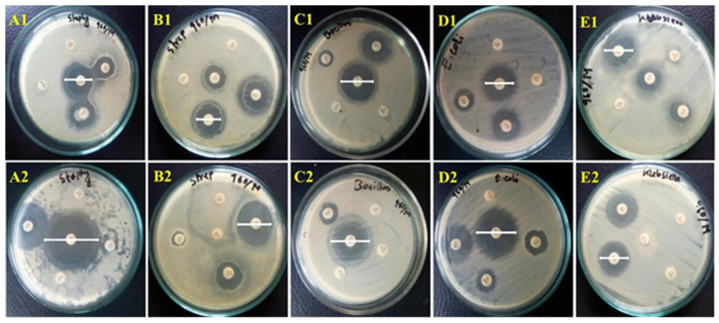
Disc diffusion test showing the zone of inhibition (↔) used to evaluate the antibiogram pattern of ten different antibiotics against specific bacteria. (**A1**,**A2**) *Staphylococcus* spp., (**B1**,**B2**) *Streptococcus* spp., (**C1**,**C2**) *Bacillus* spp., (**D1**,**D2**) *E. coli*, and (**E1**,**E2**) *Klebsiella* spp.

**Figure 5:**
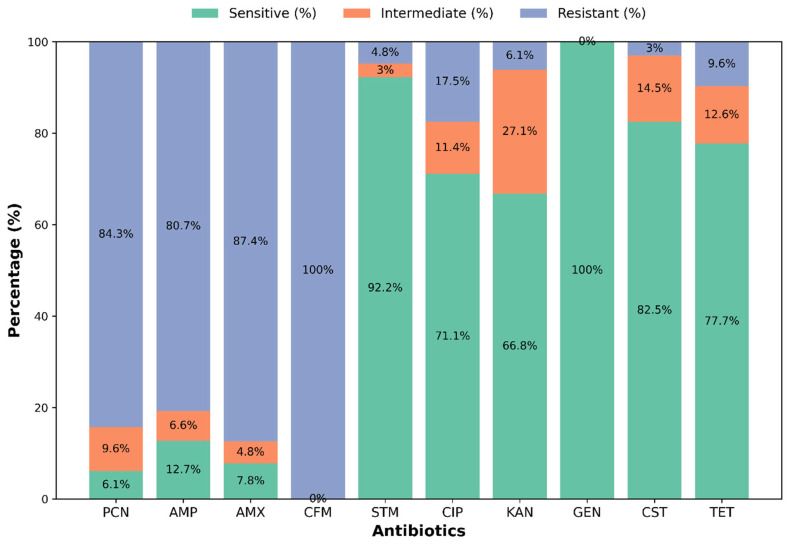
Overall antibiogram profile of the isolated bacteria. The number in the center of each color bar represents the percentage of sensitive, intermediate, and resistant bacteria. PCN, Penicillin; AMP, Ampicillin; AMX, Amoxicillin; CFM, Cefixime; STM, Streptomycin; CIP, Ciprofloxacin; KAN, Kanamycin; GEN, Gentamicin; CST, Colistin; TET, Tetracycline.

**Figure 6:**
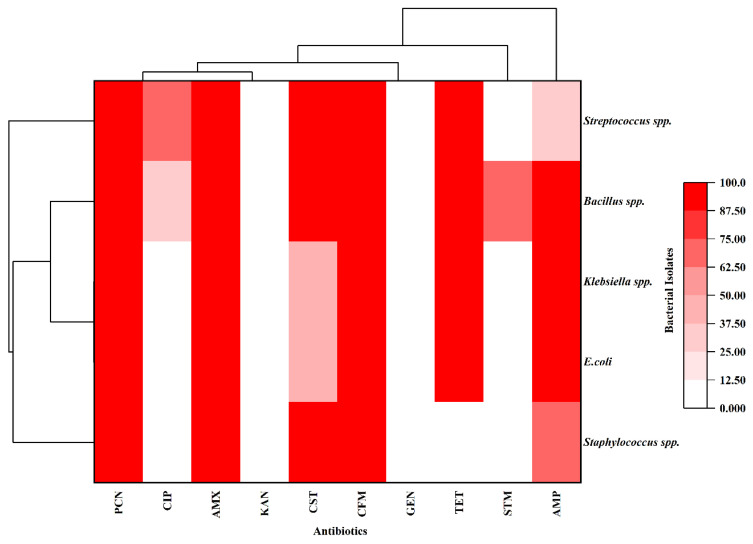
Dendrogram heatmap of the antibiogram profiles of bacterial isolates from milk. PCN, Penicillin; AMP, Ampicillin; AMX, Amoxicillin; CFM, Cefixime; STM, Streptomycin; CIP, Ciprofloxacin; KAN, Kanamycin; GEN, Gentamicin; CST, Colistin; TET, Tetracycline.

The antibiogram pattern of ten different antibiotics on specific isolates was determined using the Kirby-Bauer disc diffusion method with Muller-Hinton agar (HiMedia, India), as shown in Figure 4. Results revealed that all isolates were generally sensitive to gentamicin (GEN, 100%), streptomycin (STM, 92.2%), colistin (CST, 82.5%), tetracycline (TET, 77.7%), and ciprofloxacin (CIP, 71.1%). Intermediate sensitivity was observed for kanamycin (KAN, 27.1%), colistin (CST, 14.5%), tetracycline (TET, 12.6%), and ciprofloxacin (CIP, 11.4%). Conversely, isolates were generally resistant to ceftriaxone (CFM, 100%), amoxicillin (AMX, 87.4%), penicillin (PCN, 84.3%), and ampicillin (AMP, 80.7%) (Figure 5 and Table S5). Figure 6 and Table S6 present the heat map dendrogram of the antibiogram profile of all bacterial isolates from collected milk samples.

## 4. Discussion

This study, which included 122 lactating cows from 43 dairy herds, is among the few investigations into bovine mastitis in northern Bangladesh. The prevalence of CM and SCM was found to be 7.37% and 27.86% at the cow level, respectively. These findings are comparable to reports from Kenya (6.8%) [34] and Ethiopia (12.59%) for CM [35,36] and Bangladesh (34.2%) [28] and Sri Lanka (27.3%) for SCM [18]. However, SCM prevalence varies widely in South Asia, ranging from 20% to 80% across studies in Bangladesh, India, Pakistan, Sri Lanka [37], and Nepal [38]. The observed differences likely stem from variations in diagnostic criteria, management practices, and farm hygiene. On average, the overall prevalence reported across these studies is notably high, at around 50%. Joshi and Gokhale [39] reported lower incidence rates of CM and SCM, ranging from 1–10% and 10–50%, respectively. Although no formal power analysis was conducted prior to data collection, our sample of 122 cows across 43 farms aligns with similar epidemiological studies in resource-limited settings [24,40]. Given the logistical constraints and the prevalence studies conducted in similar regions, our sample size was determined based on practical feasibility while ensuring representative data collection.

At the quarter level, our study demonstrated an overall prevalence of 3.89% for CM and 17.82% for SCM. These results align with previous studies in Bangladesh, which reported CM rates of 5.0% [41] and SCM rates of 20.2% [23]. However, some studies found higher CM prevalence (11.6%) [7] and SCM prevalence (34.2%) [28]. This study observed a higher prevalence of CM and SCM compared to Dabele et al. [42], who reported only 1.1% prevalence of CM and 7.3% prevalence of SCM, respectively. Conversely, Abebe et al. [43] observed a lower CM prevalence (1.3%) but a higher SCM prevalence (34.7%). The observed variations in prevalence rates across studies can be attributed to the complexity of mastitis, regional differences, and the influence of various risk factors and management practices [44].

Several potential risk factors were examined. While breed and age were not statistically significant (*p* > 0.01), it is noteworthy that Friesian crosses were the only breed showing a statistically significant association (*p* < 0.05) with mastitis, indicating a markedly higher susceptibility compared to other genotypes. We observed that Sahiwal cross and local breeds had lower mastitis prevalence (12.50% and 13.33%) compared to Friesian and Jersey crossbreeds (46.57% and 27.77%). The comparatively low prevalence among Sahiwal cross and local cows likely reflects their stronger adaptation and inherent disease resilience, stronger immune competence, and is partly associated with reduced production stress. Conversely, the elevated prevalence in Friesian and Jersey crossbreds aligns with evidence that high-yielding exotic genotypes are more prone to intramammary infections due to udder conformation, metabolic load, and heightened sensitivity to environmental and management conditions. This finding aligns with earlier studies indicating that crossbred cows, due to higher milk yields and genetic predisposition, are more susceptible to mastitis [15,36]. Additionally, older cows (>9 years) and multiparous cows (≥7 parities) showed higher infection rates, consistent with previous findings, such as Chen et al. [45], who reported increased mastitis prevalence in advanced age (≥7 years) and higher parity groups. Although age was not statistically significant, cows above nine years showed a clear tendency toward higher susceptibility, likely due to the gradual weakening of teat sphincter tone and prolonged pathogen exposure. More importantly, mastitis risk increased markedly with rising parity, with cows in the 4–6 and ≥7 parity groups exhibiting a distinctly higher and statistically significant (*p* < 0.05) susceptibility. This pattern is biologically plausible, as repeated milking cycles contribute to chronic teat-end wear, reduced tissue elasticity, and compromised udder defense mechanisms [4,14,44].

Our investigation revealed a significant correlation among mastitis infection and lactation stage, high milk yield, and past disease history (*p* < 0.01). The early and late stages of lactation had higher mastitis prevalence rates, supporting the findings of Abebe et al. [43] and Fesseha et al. [46]. Physiologically, early lactation is characterized by periparturient immunosuppression, which increases susceptibility to infections [47]. The transition from pregnancy to lactation places metabolic stress on dairy cows, leading to reduced neutrophil function and impaired immune responses. In late lactation, prolonged exposure to environmental pathogens and compromised teat canal integrity contribute to higher mastitis prevalence [48,49]. These factors necessitate targeted management strategies during these critical lactation stages to mitigate mastitis risk. Additionally, in early lactation, cows are stressed by post-parturition conditions, while in late lactation stages, damage to the teat canal increases the risk of SCM by facilitating pathogen entry [37]. However, another study by Etifu and Tilahun [27] exhibited the highest mastitis prevalence (100%) in the early lactation stage, compared to those in the late (68%) and mid (43.3%) lactation stages, with a highly significant statistical difference (*p* < 0.01). In contrast, the highest prevalence of clinical mastitis was found in late and mid-lactation stages, compared to early lactation reported by Chen et al. [45]. The highest frequency occurred in high milk-yield cows, agreeing with Fesseha et al. [46], possibly due to the likelihood of injury to large-sized udders, which are more susceptible to pathogen infection [7]. Previous reports indicated a strong link between a history of disease and mastitis infection [4,34,46].

In our research, the occurrence of mastitis was statistically negligible with husbandry practices, which is generally consistent with the observations of Mbindyo et al. [34] and Fesseha et al. [46], who reported a higher incidence of mastitis in intensive farming practices (76.4% and 53.2%) compared to semi-intensive practices (65.8% and 46.8%). However, unlike their studies, where the differences were statistically significant, our findings did not reach statistical significance. Ranasinghe et al. [18] found a contrasting trend, reporting a lower incidence of mastitis in intensive farming practices (15.8%) compared to semi-intensive practices (47.4%). Mastitis occurred more frequently (78.94%) in animals on muddy floors than on concrete floors (27.18%), similar to Maalik et al. [17], Dabele et al. [42], and Kemal et al. [50] but inconsistent with Rahman et al. [4], Mbindyo et al. [34], Islam et al. [28], and Lakew et al. [51] who reported lower mastitis prevalence (8.5–28.6%) on barn floors and higher prevalence (30–76.2%) on concrete floors. However, farms that lacked udder washing before and after milking and farm hygiene practices were significantly more prone to mastitis, consistent with the findings of Maalik et al. [17], Bari et al. [37], and Dabele et al. [42] but differing from Tesfaye and Abera [52]. In addition to these hygiene practices, implementing biosecurity measures such as controlled farm access, disinfection protocols, and isolation of infected cows can further reduce mastitis prevalence, as suggested by Michigan State University Extension 2024. Daily floor cleaning and washing hands before milking result in significantly lower prevalence rates (21.65% and 25.47%) compared to weekly floor cleaning and irregular hand washing (88.0% and 100%), consistent with the findings of Abebe et al. [43] and Mbindyo et al. [34]. Additionally, the prevalence of mastitis was insignificant in cows milked by hand (35.24%) compared to those milked by machine (0%). This result aligns with the findings of Fesseha et al. [46] and Lakew et al. [51], who also reported a higher prevalence of mastitis in hand-milked cows (52.34% and 54.8%) than in machine milking. Regarding milking types, our study revealed a mastitis prevalence of 20.48% in cows subjected to complete milking, whereas the prevalence was substantially higher at 66.66% in cows subjected to partial milking, indicating a statistically significant difference. The data suggests that the presence of bedding and the practice of dry cow therapy may reduce the incidence of mastitis, although these effects are not statistically significant compared to their absence, as previously noted by Mbindyo et al. [34].

Bacteriological analysis revealed that *Staphylococcus* spp. was the most predominant organism (42%), inconsistent with Ali et al. [15] and Fesseha et al. [46], who found similar isolates contributing about 34% and 45.1% of the overall pathogens, respectively. *Streptococcus* spp. (23%) and *E. coli* (17%) were the second and third most prevalent bacteria, consistent with Mbindyo et al. [34] and Fesseha et al. [46]. These results contradicted Etifu and Tilahun [27], who found *Streptococcus* spp. at 5.8% and *E. coli* at 9.4%, respectively. However, Singha et al. [7] found that *Streptococcus* spp. (22.9%) was the most prevalent species, while *Staphylococcus aureus* (2.6%) was the least prevalent among the identified species. Our study reported a 13% incidence of *Klebsiella* spp. higher than the 5.8% and 5% incidences of *Bacillus* spp. lower than the 8.0% reported by Etifu and Tilahun [27].

According to the antibiogram study, isolated bacteria observed the highest sensitivity to gentamicin (100%), followed by streptomycin (92.2%), colistin (82.5%), tetracycline (77.7%), and ciprofloxacin (71.1%). Conversely, complete resistance was observed for cephalexin (100%), with high resistance rates for amoxicillin (87.4%), penicillin (84.3%), and ampicillin (80.7%), underscoring the growing challenge of antimicrobial resistance in bovine mastitis management. This resistance trend suggests that traditional first-line treatments may no longer be effective, necessitating a revision of treatment protocols in the region. The ineffectiveness of β-lactam antibiotics highlights the urgent need for antibiotic stewardship programs and alternative therapeutic strategies, such as targeted antimicrobial therapy based on culture and sensitivity testing [53,54]. The clinical relevance of intermediate sensitivity to ciprofloxacin lies in its potential for treatment failure in cases where bacterial loads are high or where suboptimal dosing may promote resistance development [55,56]. Intermediate susceptibility indicates that higher doses or combination therapies might be required to achieve therapeutic efficacy, necessitating careful consideration in clinical settings [57]. These findings align with Ali et al. [15], Dabele et al. [42], and Etifu and Tilahun [27], who revealed that gentamicin, ciprofloxacin, and tetracycline were most effective against bovine mastitis, whereas penicillin and ampicillin were less potent. Our investigation also agrees with Kemal et al. [49], who demonstrated that *Staphylococcus* spp. is susceptible to gentamicin (95.3%) and streptomycin (65.9%), and resistant to penicillin (87.3%), ampicillin (55.1%), and amoxicillin (53.3%). The resistance of β-lactam antibiotics to most bacterial pathogens is likely due to their extensive use in veterinary and human healthcare in this region [21].

Although this study provides valuable information on the epidemiology and antimicrobial resistance patterns of bovine mastitis in Northern Bangladesh, its cross-sectional design has limitations. Since cross-sectional studies collect data at a single time point, establishing causal relationships between risk factors and mastitis occurrence is challenging [58]. A longitudinal study design would be better suited to assess temporal trends and causality. Moreover, the lack of somatic cell count (SCC) data restricts the ability to measure the severity of subclinical mastitis. Future research should include SCC measurements and longitudinal data collection to provide a more complete understanding of mastitis dynamics in the region. Given the relatively small sample size, statistical power may be limited. Nonetheless, similar studies with comparable sample sizes have still offered valuable epidemiological insights in resource-limited settings [24]. While a larger sample would strengthen the findings, our study provides important preliminary data on mastitis prevalence, risk factors, and antibiotic resistance in Northern Bangladesh. Future studies with larger datasets are recommended to further validate these findings.

## 5. Conclusions

This study highlights the high prevalence (35.24%) of clinical and subclinical mastitis in dairy cows in Northern Bangladesh, with *Staphylococcus* spp. identified as the most common pathogen. Key risk factors contributing to mastitis included early and late lactation stages, high milk yield, previous history of mastitis, muddy floor type, poor frequency of floor cleaning, partial milking practices, and poor udder and farm hygiene. The antibiogram analysis revealed significant resistance to commonly used antibiotics such as Cefixime, Amoxicillin, Penicillin, and Ampicillin, though Gentamicin, Streptomycin, Colistin, and Tetracycline were notably effective. The widespread resistance to commonly used antibiotics underscores the necessity of responsible antibiotic use in dairy farming. Implementing stricter regulations, encouraging prudent antimicrobial application, and prioritizing preventive strategies such as better farm hygiene are crucial steps toward safeguarding both livestock health and public well-being. Given the potential impact on both animal and public health, these findings underscore the importance of implementing stricter guidelines for antibiotic use to mitigate resistance. However, this study’s findings are limited by its regional focus, cross-sectional design, and lack of somatic cell count data, which restricts a full assessment of mastitis severity. Expanding future studies to include diverse regions, longitudinal analysis trends over time, broader pathogen, climate, and environmental assessments, as well as genetic influences, would provide a more comprehensive understanding of mastitis epidemiology across Bangladesh and support the development of targeted, evidence-based interventions.

## Figures and Tables

**Table 1:** Prevalence of mastitis in dairy cows in Northern Bangladesh.

Observation	Overall Mastitis				
	Examined	Positive	Clinical Mastitis	Subclinical Mastitis	Prevalence
Cow-level	122	43	9 (7.37%)	34 (27.86%)	43 (35.24%)
Quarter-level	488	106	19 (3.89%)	87 (17.82%)	106 (21.72%)

**Table 2:** Animal-related risk factors associated with mastitis in dairy cows in Northern Bangladesh.

Variables	N	Positive	Prevalence (%)	OR (95% CI)	*p* Value
Breeds					
Sahiwal cross	16	2	12.50	Ref	
Friesian Cross	73	34	46.57	6.10 (1.29–28.79)	0.022 *
Jersey cross	18	5	27.77	2.69 (0.44–16.39)	0.282
Local	16	2	13.33	1.08 (0.13–8.80)	0.945
Age					
2–5 years	38	12	31.57	Ref	
6–9 years	61	22	36.06	1.22 (0.70–2.12)	0.909
>9 years	23	9	39.13	1.39 (0.74–2.63)	0.909
Parity					
1–3	81	22	27.16	Ref	
4–6	36	17	47.22	2.40 (1.18–4.85)	0.036 *
≥7	5	4	80.00	10.73 (4.47–25.59)	0.038 *
Lactation stage					
Early	44	21	47.72	5.74 (2.13–15.49)	0.000 **
Mid	51	7	13.72	Ref	
Late	27	15	55.55	7.86 (2.61–23.63)	0.000 **
Milk yield					
<5 L	26	5	19.23	Ref	
5–10 L	59	15	25.42	1.43 (1.00–2.06)	0.54
>10 L	37	23	62.16	6.90 (3.46–12.78)	0.000 **
Previous history of mastitis					
No	96	27	28.12	Ref	
Yes	26	16	61.53	4.10 (1.76–9.53)	0.001 **

* Significant at *p* < 0.05; ** Significant at *p* < 0.01; N, number of animals; Ref, reference category; OR, odd ratio; CI, confidence interval.

**Table 3:** Farm-related risk factors associated with mastitis in dairy cows in Northern Bangladesh.

Variables	N	Positive	Prevalence (%)	OR (95% CI)	*p* Value
Husbandry practice					
Semi-intensive	31	7	22.58	Ref	0.126
Intensive	91	36	39.56	2.24 (0.88–5.75)	
Floor type					
Mud	103	28	27.18	Ref	
Concrete	19	15	78.94	10.04 (3.07–33.86)	0.000 **
Frequency of floor cleaning					
Daily	97	21	21.65	Ref	
Weekly	25	22	88.00	26.54 (7.24–97.33)	0.000 **
Presence of bedding					
Yes	85	29	34.12	Ref	
No	37	14	37.83	0.85 (0.35–2.10)	0.785
Udder washing					
Yes	83	12	14.45	Ref	
No	39	31	79.48	17.10 (3.47–25.1)	0.000 **
Milking method					
Machine milking	0	0	0	Ref	
Hand milking	122	43	35.24	-	-
Hand washing before milking					
Yes	106	27	25.47	Ref	
No	16	16	100	-	0.000 **
Milking type					
Complete	83	17	20.48	Ref	
Partial	39	26	66.66	7.76 (6.73–36.84)	0.002 **
Teat dipping after milking					
Yes	34	7	20.59	Ref	
No	88	36	40.91	0.37 (0.14–0.55)	0.131
Dry therapy					
Yes	29	7	24.14	Ref	
No	93	36	38.71	1.99 (0.77–5.13)	0.306
Farm hygiene					
Good	87	18	20.69	Ref	
Bad	35	25	71.42	9.57 (3.91–23.36)	0.001 **

** Significant at *p* < 0.01; N, number of animals; Ref; reference category, OR, odd ratio; CI; confidence interval.

## Data Availability

The original contributions presented in this study are included in the article/Supplementary Material. Further inquiries can be directed to the corresponding author(s).

## Supplementary Materials

The following supporting information can be downloaded at: https://www.mdpi.com/article/10.3390/vetsci12121201/s1. Table S1: Farms survey questionnaire for dairy farmers. Table S2: Cultural and morphological properties of isolated bacteria. Table S3: Biochemical properties of isolated bacteria. Table S4: Frequency of occurrence of bacterial isolates from clinical and subclinical mastitis at Dinajpur, Northern Bangladesh. Table S5: Antimicrobial sensitivity profile of isolated bacteria. Table S6: Result of Antibiotic Sensitivity Test. Figure S1: Culture of isolated bacteria on Nutrient’s Agar; A = Culture of organism on Nutrient’s Agar, B = Control of Nutrient’s Agar. Figure S2: Gram Staining; A = Gram positive short chain shaped violet color *Streptococcus* spp., B = Gram positive grapes liked violet color *Staphylococcus* spp., C = Gram positive long rod of shape or chain liked violet color *Bacillus* spp., D = *Gram negative large pink color E. coli*, E = Gram negative short pink color *Klebsiella* spp. under 100x microscope. Figure S3: Cultural and morphological properties of isolated bacteria on MacConkey Agar; A = *E. coli*, B = *Klebsiella* spp., C = Control. Figure S4: Cultural and morphological properties of isolated bacteria on Eosin Methylene Blue agar; A = *E. coli*, B = *Klebsiella* spp., and C = Control. Figure S5: Cultural and morphological properties of isolated bacteria on Mannitol Salt agar; A = *Staphylococcus* spp., and B = *Streptococcus* spp. Figure S6: Cultural and morphological properties of isolated bacteria on Blood Agar; A = β-hemolytic colonies of *Staphylococcus* spp., B = β-hemolytic colonies of *Streptococcus* spp., C = hemolytic colonies of *E. coli*, D = *Bacillus* spp. with hemolysis, E = Control. Figure S7: Catalase test for biochemical properties of isolated bacteria; A = *Staphylococcus* spp., *Klebsiella* spp., and *Bacillus* spp. catalase *positive*, B = *Streptococcus* spp., and *E. coli* catalase negative. Figure S8: Citrate Test for biochemical properties of isolated bacteria; A = *Streptococcus* spp., B = *E. coli*, C = *Staphylococcus* spp., and D = *Klebsiella* spp., Tube D was positive results by changing medium green to blue coloration. Figure S9: Indole test for biochemical properties of isolated bacteria; A = *Streptococcus* spp., B = *Staphylococcus* spp., C = *Bacillus* spp., D = *E. coli*, and E = *Klebsiella* spp., Tube D was positive results indicated cherry red coloration. Figure S10: TSI test for biochemical properties of isolated bacteria; A = *Klebsiella* spp., B = *E. coli*, C = *Bacillus* spp., D = *Staphylococcus* spp. and E = *Streptococcus* spp. Tube A and B were positive results indicated yellow coloration both butt and slant. Figure S11: Methyl red test for biochemical properties of isolated bacteria; A = *Staphylococcus* spp., B = *Streptococcus* spp., C = *Bacillus* spp., D = *Klebsiella* spp., and E = *E. coli*. Tube A, C and E were positive results indicated red coloration. Figure S12: Voges-Proskauer test for biochemical properties of isolated bacteria. A = *Streptococcus* spp., B= *Staphylococcus* spp., C = *Bacillus* spp., D = *Klebsiella* spp., and E = *E. coli*. Tube B, C and D were positive results indicated red coloration. Figure S13: Starch test for biochemical properties of isolated bacteria; A = Control, B = *Staphylococcus* spp., C = *E. coli*, D = *Klebsiella* spp., E = *Bacillus* spp., and F = *Streptococcus* spp. *Bacillus* spp. (tube E) don’t farment suger that indicated positive results.

## References

[1] Banglapedia—National Encyclopedia of Bangladesh. Livestock. https://en.banglapedia.org/index.php?title=Livestock.

[2] DLS Livestock-Economy—Department of Livestock Services. http://www.dls.gov.bd/site/page/22b1143b-9323-44f8-bfd8-647087828c9b/Livestock-Economy,%202021.

[3] Shamsuddoha A., Edwards G.W. Dairy industry in Bangladesh: Problems and prospects. Proceedings of the Australian Agricultural and Resource Economics Society (AARES).

[4] Rahman M., Bhuiyan M., Kamal M., Shamsuddin M. (2009). Prevalence and risk factors of mastitis in dairy cows. Bangladesh Vet..

[5] Sayeed M.A., Rahman M.A., Bari M.S., Islam A., Rahman M.M., Hoque M.A. (2020). Prevalence of subclinical mastitis and associated risk factors at cow level in dairy farms in Jhenaidah, Bangladesh. Adv. Anim. Vet. Sci..

[6] Hogeveen H., Huijps K., Lam T. (2011). Economic aspects of mastitis: New developments. N. Zealand Vet. J..

[7] Singha S., Koop G., Persson Y., Hossain D., Scanlon L., Derks M., Hoque M.A., Rahman M.M. (2021). Incidence, etiology, and risk factors of clinical mastitis in dairy cows under semi-tropical circumstances in Chattogram, Bangladesh. Animals.

[8] Azooz M.F., El-Wakeel S.A., Yousef H.M. (2020). Financial and economic analyses of the impact of cattle mastitis on the profitability of Egyptian dairy farms. Vet. World.

[9] Rollin E., Dhuyvetter K.C., Overton M.W. (2015). The cost of clinical mastitis in the first 30 days of lactation: An economic modeling tool. Prev. Vet. Med..

[10] Glasgow U.O. Potential Biomarkers of Mastitis in Dairy Cattle Milk Identified. https://phys.org/news/2016-07-potential-biomarkers-mastitis-dairy-cattle.html.

[11] Kibebew K. (2017). Bovine mastitis: A review of causes and epidemiological point of view. J. Biol. Agric. Healthc..

[12] Cheng W.N., Han S.G. (2020). Bovine mastitis: Risk factors, therapeutic strategies, and alternative treatments—A review. Anim. Biosci..

[13] Quinn P.J., Markey B.K., Leonard F.C., Hartigan P., Fanning S., Fitzpatrick E. (2011). Veterinary Microbiology and Microbial Disease.

[14] Abebe R., Markos A., Abera M., Mekbib B. (2023). Incidence rate, risk factors, and bacterial causes of clinical mastitis on dairy farms in Hawassa City, southern Ethiopia. Sci. Rep..

[15] Ali T., Kamran, Raziq A., Wazir I., Ullah R., Shah P., Ali M.I., Han B., Liu G. (2021). Prevalence of mastitis pathogens and antimicrobial susceptibility of isolates from cattle and buffaloes in Northwest of Pakistan. Front. Vet. Sci..

[16] Sharma D., Kaniamuthan S., Manimaran A., Kumaresan A., Sivaram M., Rajendran D., Wankhade P.R., Sejian V., Banu S. (2023). Seasonal, physiological and bacteriological risk factors for subclinical mastitis in dairy cows maintained under different farming conditions. J. Dairy Res..

[17] Maalik A., Ali S., Iftikhar A., Rizwan M., Ahmad H., Khan I. (2019). Prevalence and antibiotic resistance of *Staphylococcus aureus* and risk factors for bovine subclinical mastitis in District Kasur, Punjab, Pakistan. Pak. J. Zool..

[18] Ranasinghe R.M.S.B., Deshapriya R., Abeygunawardana D., Rahularaj R., Dematawewa C. (2021). Subclinical mastitis in dairy cows in major milk-producing areas of Sri Lanka: Prevalence, associated risk factors, and effects on reproduction. J. Dairy Sci..

[19] Gunawardana S., Thilakarathne D., Abegunawardana I.S., Abeynayake P., Robertson C., Stephen C. (2014). Risk factors for bovine mastitis in the Central Province of Sri Lanka. Trop. Anim. Health Prod..

[20] Bhandari S., Subedi D., Tiwari B.B., Shrestha P., Shah S., Al-Mustapha A.I. (2021). Prevalence and risk factors for multidrug-resistant Escherichia coli isolated from subclinical mastitis in the western Chitwan region of Nepal. J. Dairy Sci..

[21] Michira L., Kagira J., Maina N., Waititu K., Kiboi D., Ongera E., Ngotho M. (2023). Prevalence of subclinical mastitis, associated risk factors and antimicrobial susceptibility pattern of bacteria isolated from milk of dairy cattle in Kajiado Central sub-county, Kenya. Vet. Med. Sci..

[22] Oliveira C.S.F., Hogeveen H., Botelho A.M., Maia P.V., Coelho S.G., Haddad J.P.A. (2015). Cow-specific risk factors for clinical mastitis in Brazilian dairy cattle. Prev. Vet. Med..

[23] Sarker S.C., Parvin M.S., Rahman A.A., Islam M.T. (2013). Prevalence and risk factors of subclinical mastitis in lactating dairy cows in north and south regions of Bangladesh. Trop. Anim. Health Prod..

[24] Al Emon A., Hossain H., Chowdhury M.S.R., Rahman M.A., Tanni F.Y., Asha M.N., Akter H., Hossain M.M., Islam M.R., Rahman M.M. (2024). Prevalence, antimicrobial susceptibility profiles and resistant gene identification of bovine subclinical mastitis pathogens in Bangladesh. Heliyon.

[25] Gelalcha B.D., Agga G.E., Dego O.K. (2021). Antimicrobial usage for the management of mastitis in the USA: Impacts on antimicrobial resistance and potential alternative approaches. Veterinary Medicine—A Textbook of the Diseases of Cattle, Horses, Sheep, Pigs and Goats.

[26] Sharma C., Rokana N., Chandra M., Singh B.P., Gulhane R.D., Gill J.P.S., Ray P., Puniya A.K., Panwar H. (2018). Antimicrobial resistance: Its surveillance, impact, and alternative management strategies in dairy animals. Front. Vet. Sci..

[27] Melesse E., Minyahil T. (2019). Prevalence of bovine mastitis, risk factors, isolation and anti-bio gram of major pathogens in Mid Rift valley, Ethiopia. Int. J. Livest. Prod..

[28] Islam S., Barua S.R., Islam A., Moni S.P., Uddin H., Ferdous J., Rahman M.K., Hassan M.M., Rahman A.A., Chawdhury S. (2019). Epidemiology of sub-clinical mastitis in dairy cows in urban areas of Chittagong, Bangladesh. Turk. J. Agric.-Food Sci. Technol..

[29] Banglapedia—National Encyclopedia of Bangladesh. Dinajpur District. https://en.banglapedia.org/index.php?title=Dinajpur_District.

[30] Haque M.A., Wang F., Chen Y., Hossen F., Islam M.A., Hossain M.A., Siddique N., He C., Ahmed F. (2022). *Bacillus* spp. contamination: A novel risk originated from animal feed to human food chains in south-eastern Bangladesh. Front. Microbiol..

[31] Hogan J., Gonzalez R., Harmon R., Nickerson S., Oliver S., Pankey J., Smith K.L. (1999). Laboratory handbook on bovine mastitis. Natl. Mastit. Counc. Madison WI.

[32] NMC (2017). Laboratory Handbook on Bovine Mastitis.

[33] CLSI (2022). Performance Standards for Antimicrobial Susceptibility Testing.

[34] Mbindyo C.M., Gitao G.C., Mulei C.M. (2020). Prevalence, etiology, and risk factors of mastitis in dairy cattle in Embu and Kajiado Counties, Kenya. Vet. Med. Int..

[35] Girma A., Tamir D. (2022). Prevalence of bovine mastitis and its associated risk factors among dairy cows in Ethiopia during 2005–2022: A systematic review and meta-analysis. Vet. Med. Int..

[36] Tezera M., Aman Ali E. (2021). Prevalence and associated risk factors of Bovine mastitis in dairy cows in and around Assosa town, Benishangul-Gumuz Regional State, Western Ethiopia. Vet. Med. Sci..

[37] Bari M.S., Rahman M.M., Persson Y., Derks M., Sayeed M.A., Hossain D., Singha S., Hoque M.A., Sivaraman S., Fernando P. (2022). Subclinical mastitis in dairy cows in south-Asian countries: A review of risk factors and etiology to prioritize control measures. Vet. Res. Commun..

[38] Sah K., Karki P., Shrestha R.D., Sigdel A., Adesogan A.T., Dahl G.E. (2020). MILK Symposium review: Improving control of mastitis in dairy animals in Nepal. J. Dairy Sci..

[39] Joshi S., Gokhale S. (2006). Status of mastitis as an emerging disease in improved and periurban dairy farms in India. Ann. N. Y. Acad. Sci..

[40] Islam M.M., Hossain M.I., Islam M.S., Azam M.G., Sultana S. (2025). Prevalence, antibiotic resistance patterns, and virulence factors of *Staphylococcus aureus* isolates associated with bovine mastitis in northern Bangladesh. Heliyon.

[41] Islam S., Islam M.K., Khan M.R., Al-Maruf M., Zabed M.A., Mohanta U.K., Ali M.Z., Islam K.B.M.S. (2024). Prevalence of mastitis and antimicrobial resistance patterns of Escherichia coli and *Staphylococcus aureus* isolated from the infected udder of dairy cows in coastal regions. Bangladesh Vet..

[42] Dabele D.T., Borena B.M., Admasu P., Gebremedhin E.Z., Marami L.M. (2021). Prevalence and risk factors of mastitis and isolation, identification and antibiogram of *Staphylococcus* species from mastitis positive zebu cows in toke Kutaye, Cheliya, and Dendi districts, west Shewa zone, Oromia, Ethiopia. Infect. Drug Resist..

[43] Abebe R., Hatiya H., Abera M., Megersa B., Asmare K. (2016). Bovine mastitis: Prevalence, risk factors and isolation of *Staphylococcus aureus* in dairy herds at Hawassa milk shed, South Ethiopia. BMC Vet. Res..

[44] Radostits O., Gay C., Hinchcliff K., Constable P. (2007). Diseases of the mammary gland. Veterinary Medicine—A Textbook of the Diseases of Cattle, Horses, Sheep, Pigs and Goats.

[45] Chen S., Zhang H., Zhai J., Wang H., Chen X., Qi Y. (2023). Prevalence of clinical mastitis and its associated risk factors among dairy cattle in mainland China during 1982–2022: A systematic review and meta-analysis. Front. Vet. Sci..

[46] Fesseha H., Mathewos M., Aliye S., Wolde A. (2021). Study on prevalence of bovine mastitis and associated risk factors in dairy farms of Modjo town and suburbs, central Oromia, Ethiopia. Vet. Med. Res. Rep..

[47] Aleri J.W., Hine B.C., Pyman M.F., Mansell P.D., Wales W.J., Mallard B., Fisher A.D. (2016). Periparturient immunosuppression and strategies to improve dairy cow health during the periparturient period. Res. Vet. Sci..

[48] Neculai-Valeanu A.S., Ariton A.M. (2022). Udder Health Monitoring for Prevention of Bovine Mastitis and Improvement of Milk Quality. Bioengineering.

[49] Khasapane N.G., Byaruhanga C., Thekisoe O., Nkhebenyane S.J., Khumalo Z.T.H. (2023). Prevalence of subclinical mastitis, its associated bacterial isolates and risk factors among cattle in Africa: A systematic review and meta-analysis. BMC Vet. Res..

[50] Kemal K.E., Tesfaye S., Ashanafi S., Muhammadhussien A.F. (2017). Prevalence, risk factors and multidrug resistance profile of *Staphylococcus aureus* isolated from bovine mastitis in selected dairy farms in and around Asella town, Arsi Zone, South Eastern Ethiopia. Afr. J. Microbiol. Res..

[51] Lakew B.T., Fayera T., Ali Y.M. (2019). Risk factors for bovine mastitis with the isolation and identification of Streptococcus agalactiae from farms in and around Haramaya district, eastern Ethiopia. Trop. Anim. Health Prod..

[52] Tesfaye B., Abera A. (2018). Prevalence of mastitis and associated risk factors in Jimma town dairy farms, Western Ethiopia. J. Vet. Sci. Anim. Husb..

[53] Muteeb G., Rehman M.T., Shahwan M., Aatif M. (2023). Origin of Antibiotics and Antibiotic Resistance, and Their Impacts on Drug Development: A Narrative Review. Pharmaceuticals.

[54] Yamin D., Uskoković V., Wakil A.M., Goni M.D., Shamsuddin S.H., Mustafa F.H., Alfouzan W.A., Alissa M., Alshengeti A., Almaghrabi R.H. (2023). Current and Future Technologies for the Detection of Antibiotic-Resistant Bacteria. Diagnostics.

[55] Udy A.A., Roberts J.A., Lipman J. (2013). Clinical implications of antibiotic pharmacokinetic principles in the critically ill. Intensive Care Med..

[56] Shariati A., Arshadi M., Khosrojerdi M.A., Abedinzadeh M., Ganjalishahi M., Maleki A., Heidary M., Khoshnood S. (2022). The resistance mechanisms of bacteria against ciprofloxacin and new approaches for enhancing the efficacy of this antibiotic. Front. Public Health.

[57] Rodloff A., Bauer T., Ewig S., Kujath P., Müller E. (2008). Susceptible, intermediate, and resistant—The intensity of antibiotic action. Dtsch. Arztebl. Int..

[58] Muhwezi R., Emmanuel E., Sankarapandian V., Neel R.G., Shabohurira A., Makeri D. (2025). A systematic review and meta-analysis on the prevalence and associated factors, bacterial profiles, and antibiotic susceptibility of subclinical mastitis in dairy cattle in Uganda. Sci. Afr..

