# Supplementary material for: Prevalence, Risk Factors, and Antibiogram Analysis of Bovine Mastitis in Northern Bangladesh
Source: Vet Sci. 2025 Dec 15;12(12):1201. doi: 10.3390/vetsci12121201 (PMC12737437; doi:10.3390/vetsci12121201)
Supplement: Supplementary file 1 [file vetsci-12-01201-s001.zip › vetsci-3993973-supplementary.pdf]

Table S1. Farms survey questionnaire for dairy farmers.

To be filled once for each farm

Sample ID:

**Demographic information**	
Name of the farm	
Owner	
Gender	
Age	
Mobile no.	
Address	

Animal related factors:

1. Breed

a) Local	b) Friesian cross	c) Jersey cross	d) Sahiwal cross

2. Age

a) 2-5 years	b) 6-9 years	c) >9 years

3. Parity

a) 1-3	b) 4-6	c) ≥7

4. Lactation stage

a) Early	b) Mid	c) Late

5. Milk yield

a) <5 liter	b) 5-10 liter	c) >10 liter

6. Mastitis history

a) Yes	b) No

Farm related factors

1. Husbandry practice

a) Intensive	b) Semi-intensive

2. Floor type

a) Mud	b) Concrete

3. How often the floor cleaned?

a) Daily	b) Weekly	c) Monthly	d) Others (specify)

4. Presence of bedding

a) Yes	b) No

5. Milking method

a) Hand milking	b) Machine milking

6. Udder washing before milking

a) Yes	b) No

7. Hand washing before milking

a) Yes	b) No

8. Milking type

a) Complete	b) Part time

9. Teat dip after milking

a) Yes	b) No

10. Dry cow therapy

a) Yes	b) No

11. Farm hygiene practice

a) Good	b) Poor

12. Who treats your animal?

a) Veterinary Surgeon	b) Animal Health Assistant	c) Self	d) Others (specify)

13. Do they perform any test to confirm mastitis?

a) CMT	b) Alcohol test	Culture	d) Others (specify)

Cow characteristics details

Cow ID	Breed	Parity	Milk production per day	Stage of lactation	History of mastitis	CMT result
Cow1						
Cow2						
Cow3						

Signature of the investigator

Date: ……………………………

Note: If necessary Bengali version of the questionnaire will be used while interviewing farm owner.

Table S2. Cultural and morphological properties of isolated bacteria.

Name of bacteria	Staining characteristics	Cultural characteristics	
		Name of media	Colony morphology
Staphylococcus spp.	Gram positive cocci shaped arranged in grape like cluster, violet color	NA	Circular, small, smooth, convex and gray-white or yellowish colonies
		MSA	Golden yellow pigmented or pale yellowish colonies
		SAN110	Pale opaque or golden yellowish colonies
		BA	ß-hemolytic colonies
Streptococcus spp.	Gram positive cocci shaped arranged in short chain, violet color	NA	Circular, small smooth, convex, and golden, yellowish colonies
		MSA	Pale yellowish colonies
		BA	ß-hemolytic colonies
E. coli	Gram negative rod shaped arranged in single, pairs or short chain, pink color	NA	Large, mucoid, circular, low convex, grayish, white colonies
		MAC agar	Large mucoid rose-pink smooth colonies
		EMB agar	Smooth circular, black color colonies with green metallic sheen
		BGA	Yellow-green colonies
Klebsiella spp.	Gram negative rod shaped, arranged in single, pairs or short chain, pink color	NA	Large circular, smooth, convex colonies
		MAC agar	Round, pink, slightly raised translucent and mucoid colonies
		EMB agar	Round, pink, slightly raised translucent and mucoid colonies
Bacillus spp.	Gram-positive large rod shaped arranged in chain	NA	Thick, grayish white or cream color colonies
		BA	Large, creamy colonies with ß hemolysis

NA = Nutrient Agar; MSA = Mannitol Salt Agar; SAN 110 = Staphylococcus Agar No. 110; BA = Blood Agar; MAC = Mac-Conkey's; EMB = Eosin Methylene Blue; BGA = Brilliant Green Agar.

Table S3. Biochemical properties of isolated bacteria.

Biochemical test	*Staphylococcus* spp.	*Streptococcus* spp.	*E. coli*	*Klebsiella* spp.	*Bacillus* spp.
SC	-	-	-	+	+
IT	-	-	+	-	-
TSI	S- Yellowish with colony, B- No change, Gas -, H2S -	S- Pinkish with colony, B- No change, Gas -, H2S -	S- Yellowish with colony, B- No change, Gas +, H2S -	S- Yellowish with colony, B- No change, Gas +, H2S -	S- Pinkish with colony, B- No change, Gas -, H2S -
MR	+	-	+	-	+
VP	+	-	-	+	+
C	+	-	-	+	+
LA	-	-	-	-	+
SA	-	-	-	-	+

SC= Simon Citrate test; IT= Indole test; TSI= Triple sugar iron test; MR= Methyl-Red test; VP= Voges-Proskauer test; C= Catalase test; LA= Lecithinase activity; S= Starch activity; S= Slant; B= Butt; + = Positive reaction; - = Negative reaction.

Table S4. Frequency of occurrence of bacterial isolates from clinical and subclinical mastitis at Dinajpur, Northern Bangladesh.

Bacteria	Clinical mastitis (%)	Sub-clinical mastitis (%)	Total Prevalence (%)
Staphylococcus spp.	18 (40)	51 (42.1)	69 (42)
Streptococcus spp.	11 (24.4)	28 (23.1)	39 (23)
Escherichia coli	8 (17.7)	21 (17.3)	29 (17)
Klebsiella spp.	5 (11.1)	16 (13.2)	21 (13)
Bacillus spp.	3 (6.6)	5 (4.1)	8 (5)
Total	45 (27.1)	121 (72.8)	166 (100)

Table S5. Antimicrobial sensitivity profile of isolated bacteria.

Antibiotic	Sensitive (%)	Intermediate (%)	Resistant (%)
Penicillin	10 (6.1)	16 (9.6)	140 (84.3)
Ampicillin	21 (12.7)	11 (6.6)	134 (80.7)
Amoxicillin	13 (7.8)	8 (4.8)	145 (87.4)
Cefixime	0 (0)	0 (0)	166 (100)
Streptomycin	153 (92.2)	5 (3)	8 (4.8)
Ciprofloxacin	118 (71.1)	18 (11.4)	29 (17.5)
Kanamycin	111 (66.8)	45 (27.1)	10 (6.1)
Gentamicin	166 (100)	0 (0)	0 (0)
Colistin	137 (82.5)	24 (14.5)	5 (3)
Tetracycline	129 (77.7)	21 (12.6)	16 (9.6)

PCN=Penicillin, AMP= Ampicillin, AMX=Amoxicillin, CFM=Cefixime, STM=Streptomycin, CIP=Ciprofloxacin, KAN=Kanamycin, GEN=Gentamicin, CST=Colistin, TET=Tetracycline.

Table S6: Result of Antibiotic Sensitivity Test.

Antibacterial agents	*Staphylococcus spp* (69)			*Streptococcus spp* (39)			*Bacillus spp* (8)			*E. coli* (29)			*Klebsiella spp* (21)		
	S	Int	R	S	Int	R	S	Int	R	S	Int	R	S	Int	R
Ampicilin	0	7	19	0	11	4	0	1	2	0	0	11	0	0	8
	0%	26.93%	73.07%	0%	73.33%	26.67%	0%	33.3%	66.7%	0%	0%	100%	0%	0%	100%
Ciprofloxacin	69	0	0	0	5	10	0	2	1	11	0	0	8	0	0
	100%	0%	0%	0%	33.33%	66.67%	0%	66.67%	33.33%	100%	0%	0%	100%	0%	0%
Amoxicillin	0	0	18	0	0	15	0	0	3	0	0	11	0	0	8
	0%	0%	100%	0%	0%	100%	0%	0%	100%	0%	0%	100%	0%	0%	100%
Gentamycin	69	0	0	15	0	0	3	0	0	8	0	3	8	0	0
	100%	0%	0%	100%	0%	0%	100%	0%	0%	72.3%	0%	27.3%	100%	0%	0%
Streptomycin	69	0	0	15	0	0	0	1	2	9	2	0	8	0	0
	100%	0%	0%	100%	0%	0%	0%	33.33%	66.67%	81.81%	18.19%	0%	100%	0%	0%
Cefixime	0	0	26	0	2	13	0	0	3	0	0	11	0	0	8
	0%	0%	100%	0%	13.3%	86.7%	0%	0%	100%	0%	0%	100%	0%	0%	100%
Kanamycin	18	0	0	15	0	0	0	3	0	11	0	0	8	0	0
	100%	0%	0%	100%	0%	0%	0%	100%	0%	100%	0%	0%	100%	0%	0%
Penicillin	0	0	18	0	1	14	0	0	3	0	0	11	0	0	8
	0%	0%	100%	0%	6.7%	93.3%	0%	0%	100%	0%	0%	100%	0%	0%	100%
Colistin	0	0	18	0	0	15	0	0	3	0	2	9	0	5	3
	0%	0%	100%	0%	0%	100%	0%	0%	100%	0%	18.19%	81.81%	0%	62.5%	37.5%
Tetracycline	52	0	0	0	10	5	0	1	2	0	0	11	0	0	8
	100%	0%	0%	0%	66.7%	33.3%	0%	33.3%	66.7%	0%	0%	100%	0%	0%	100%

S= Sensitive, Int= Intermediate, R= Resistant

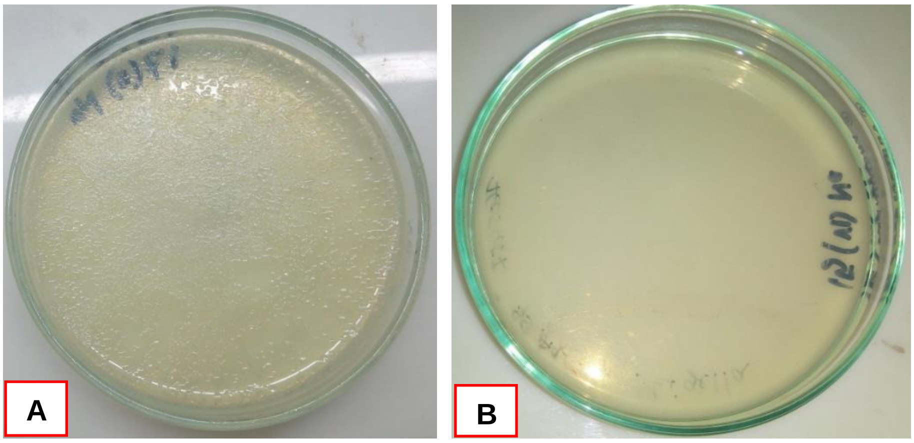

Figure S1. Culture of isolated bacteria on Nutrient’s Agar; A= Culture of organism on Nutrient’s Agar, B= Control of Nutrient’s Agar.

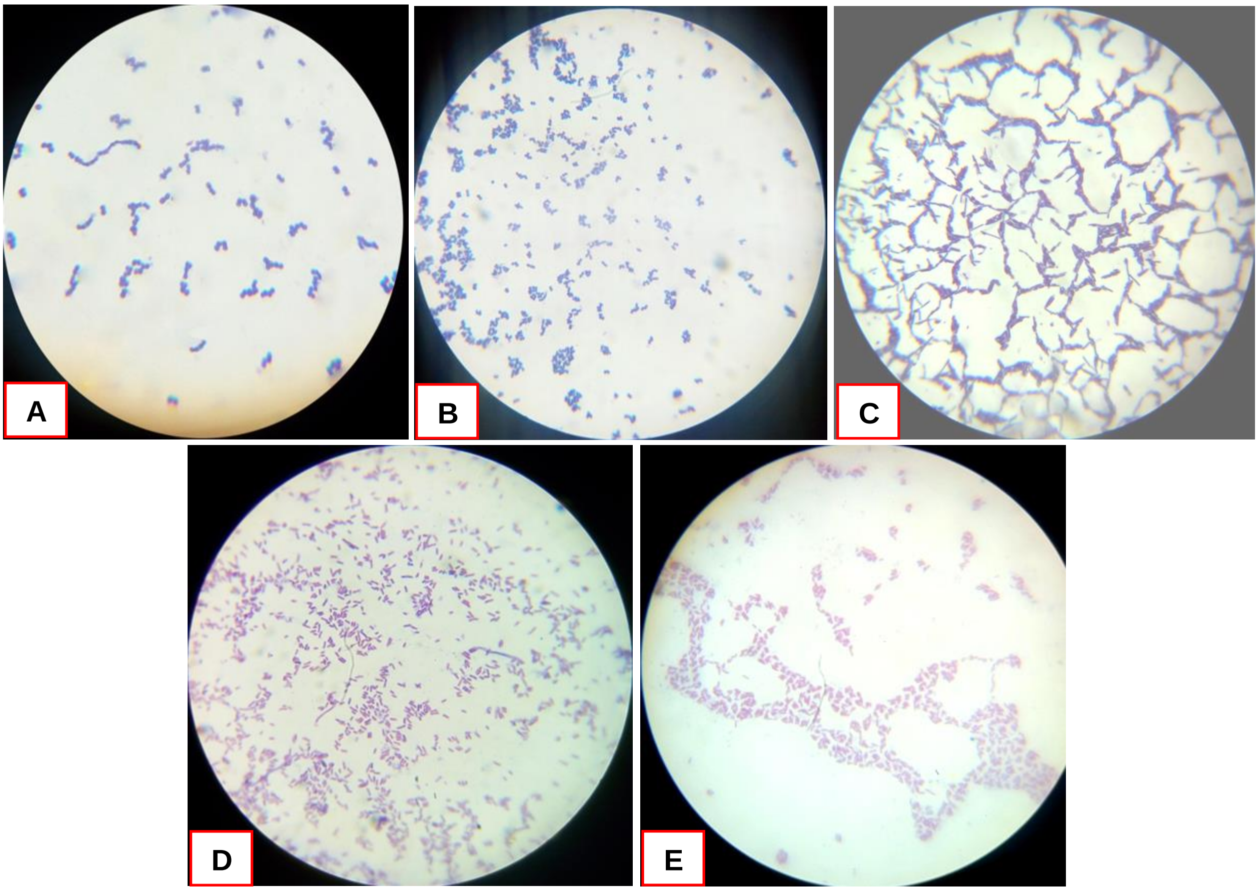

Figure S2. Gram Staining; A= Gram positive short chain shaped violet color *Streptococcus spp.*, B= Gram positive grapes liked violet color *Staphylococcus spp.*, C= Gram positive long rod of shape or chain liked violet color *Bacillus spp*., D= *Gram negative large pink color E. coli*, E= Gram negative short pink color *Klebsiella spp.* under 100x microscope.

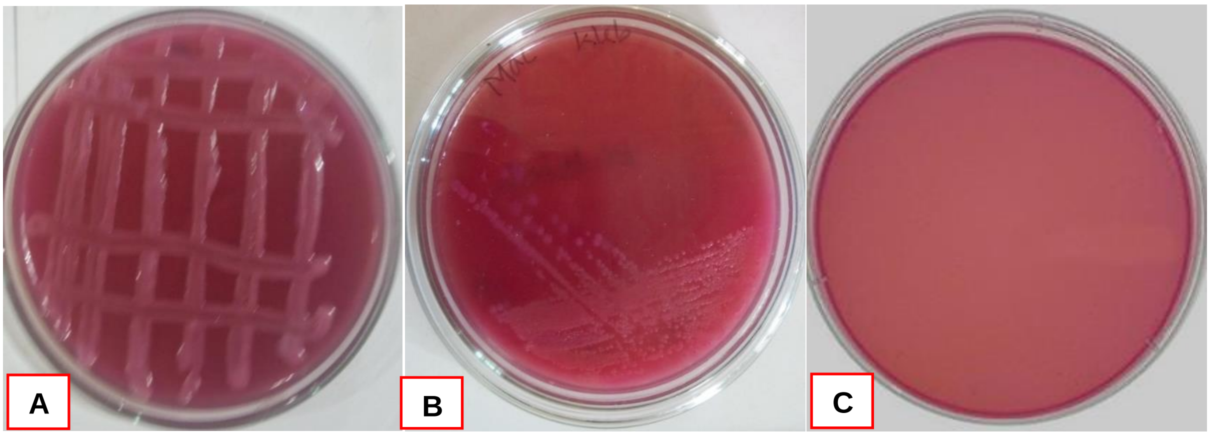

Figure S3: Cultural and morphological properties of isolated bacteria on MacConkey Agar; A= *E. coli*, B= *Klebsiella spp*., C= Control.

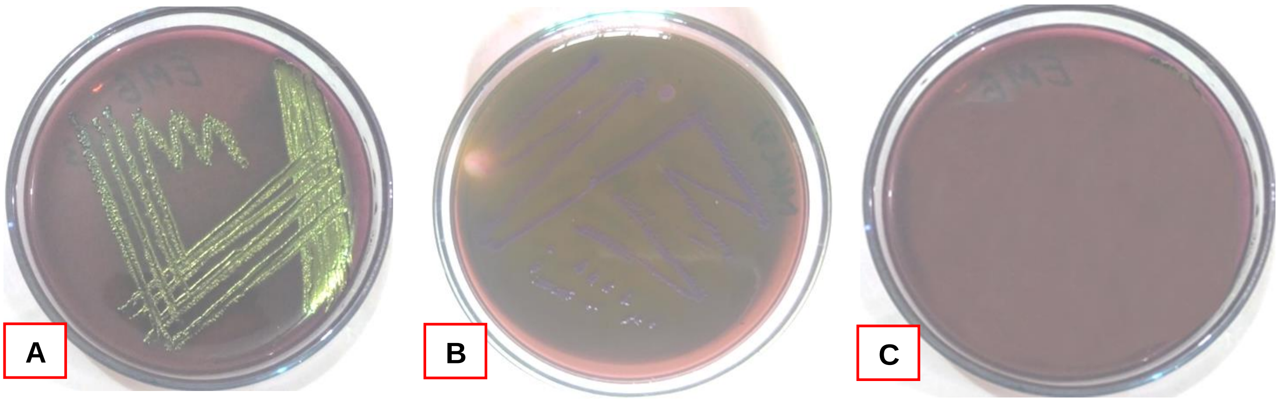

Figure S4: Cultural and morphological properties of isolated bacteria on Eosin Methylene Blue agar; A=*E. coli*, B= *Klebsiella spp.*, and C= Control.

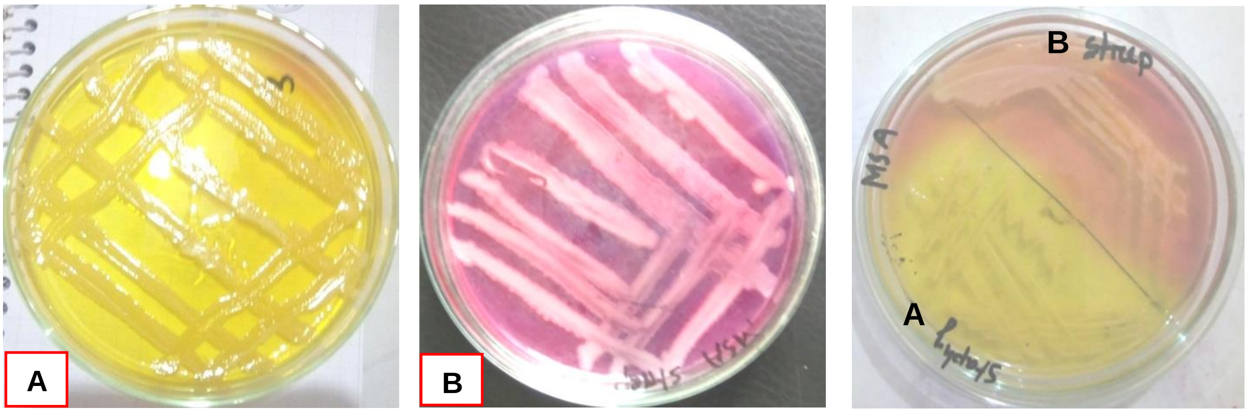

Figure S5: Cultural and morphological properties of isolated bacteria on Mannitol Salt agar; A= *Staphylococcus spp.*, and B= *Streptococcus spp.*

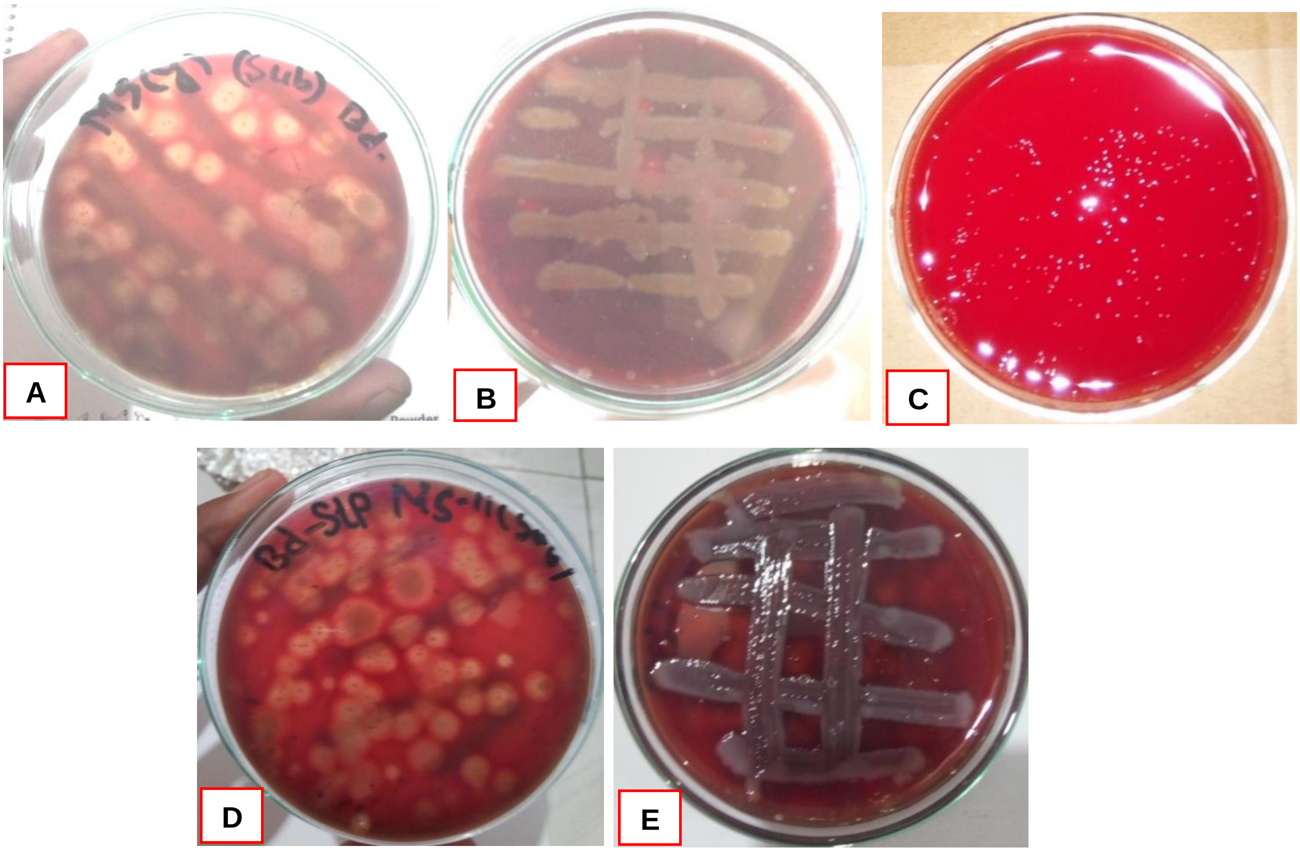

Figure S6: Cultural and morphological properties of isolated bacteria on Blood Agar; A= β-hemolytic colonies of *Staphylococcus spp*., B= β-hemolytic colonies of *Streptococcus spp.*, C= hemolytic colonies of *E. coli*, D= *Bacillus spp*. with hemolysis, E= Control.

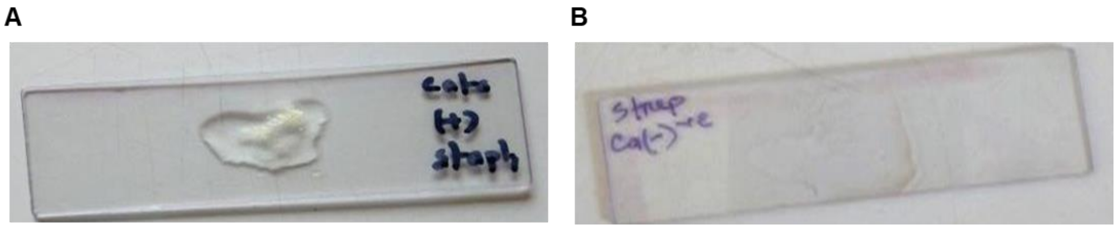

Figure S7: Catalase test for biochemical properties of isolated bacteria; A= *Staphylococcus spp., Klebsiella spp., and Bacillus spp.* catalase *positive,* B= *Streptococcus spp.,* and *E. coli* catalase negative

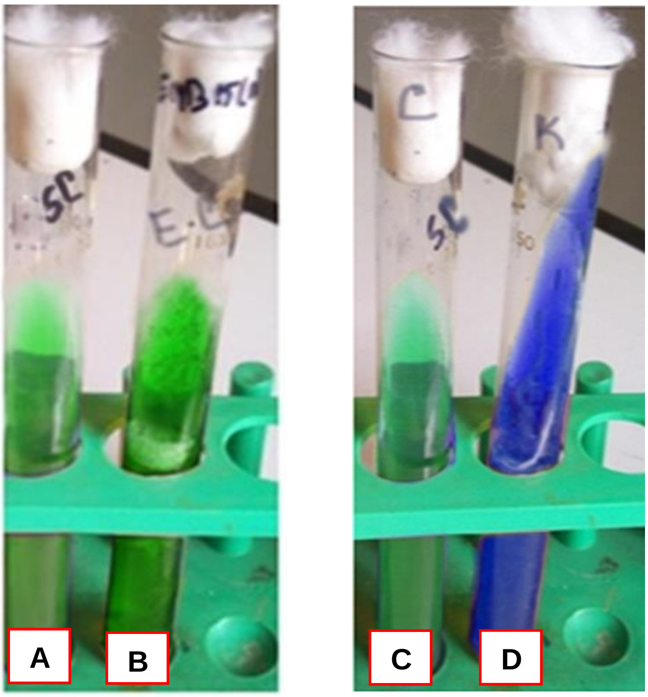

Figure S8: Citrate Test for biochemical properties of isolated bacteria; A= *Streptococcus spp.*, B= *E. coli*, C= *Staphylococcus spp.*, and D= *Klebsiella spp.*, Tube D was positive results by changing medium green to blue coloration.

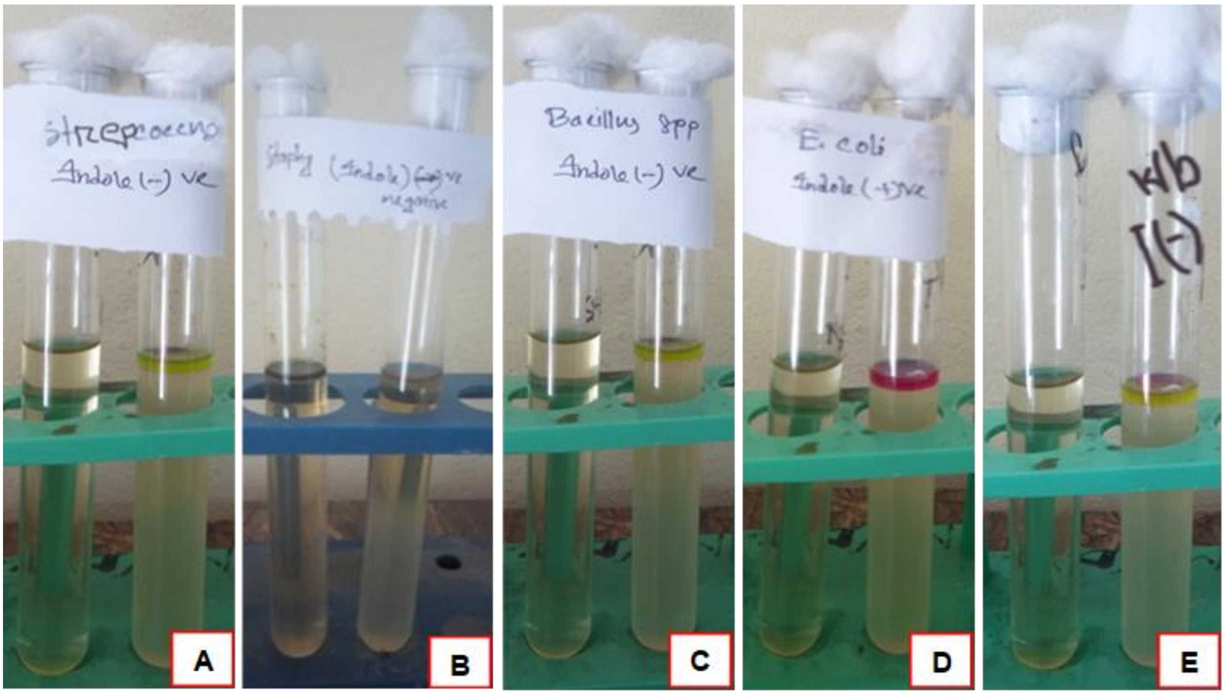

Figure S9: Indole test for biochemical properties of isolated bacteria; A= *Streptococcus spp.*, B= *Staphylococcus spp.*, C= *Bacillus spp.*, D= *E. coli*, and E= *Klebsiella spp.*, Tube D was positive results indicated cherry red coloration.

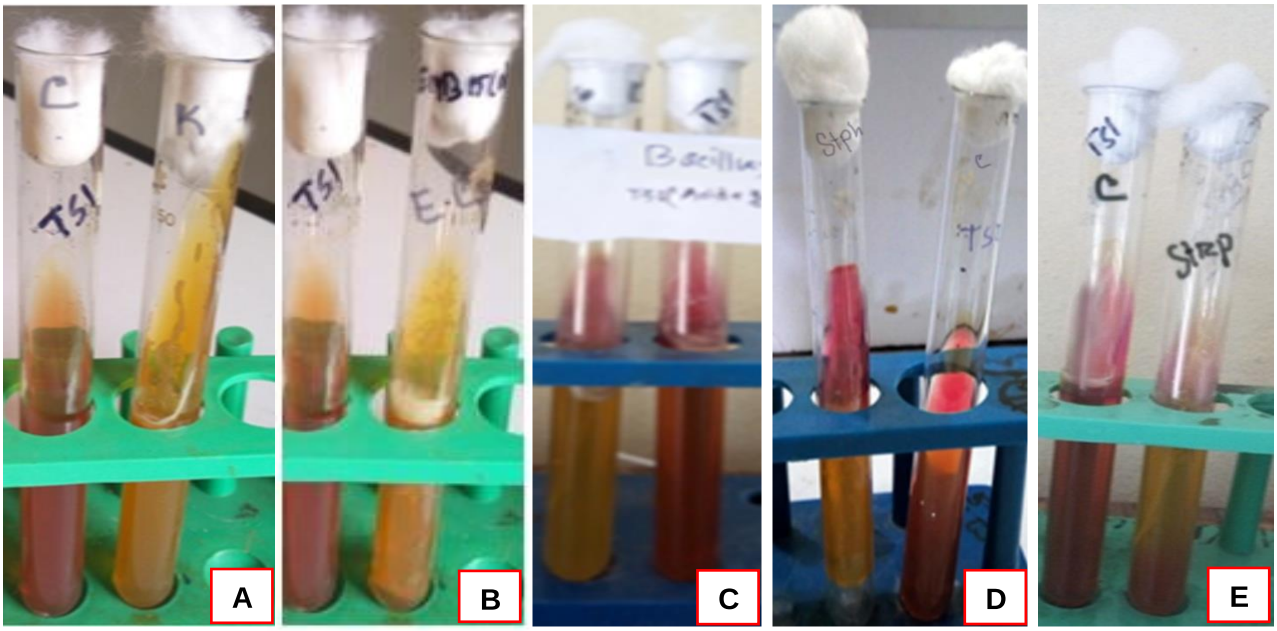

Figure S10: TSI test for biochemical properties of isolated bacteria; A= *Klebsiella spp.*, B= *E. coli*, C= *Bacillus spp.*, D= *Staphylococcus spp.* and E= *Streptococcus spp.* Tube A and B were positive results indicated yellow coloration both butt and slant.

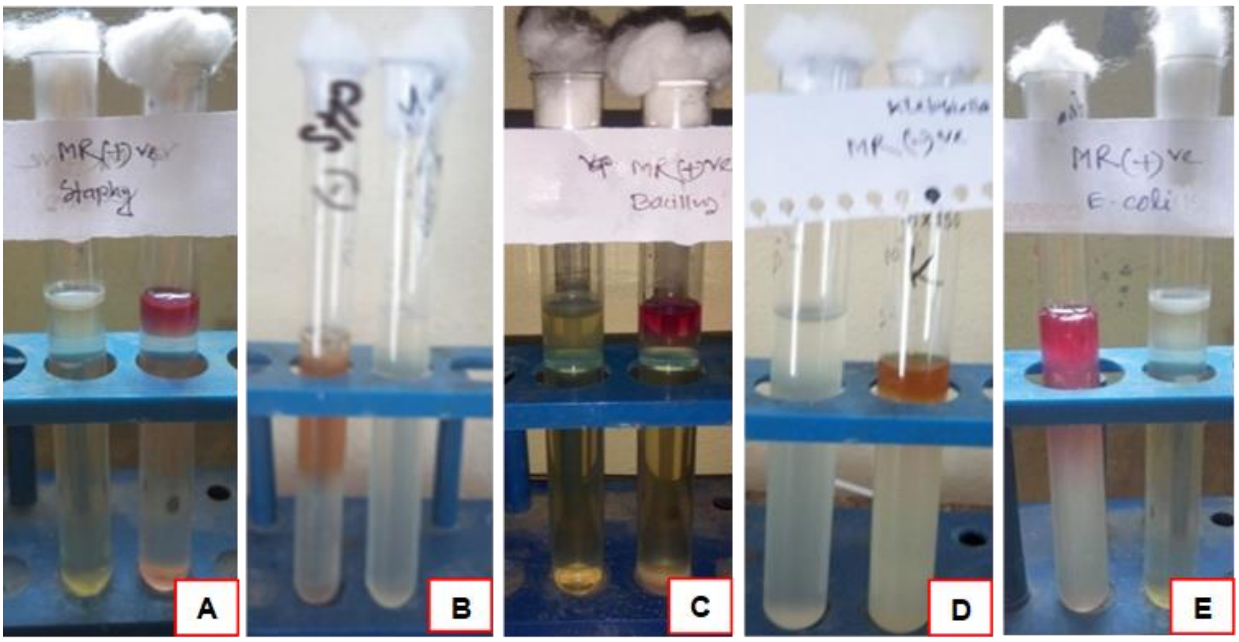

Figure S11: Methyl red test for biochemical properties of isolated bacteria; A= *Staphylococcus spp.*, B= *Streptococcus spp.*, C= *Bacillus spp.*, D= *Klebsiella spp.*, and E= *E. coli*. Tube A, C and E were positive results indicated red coloration.

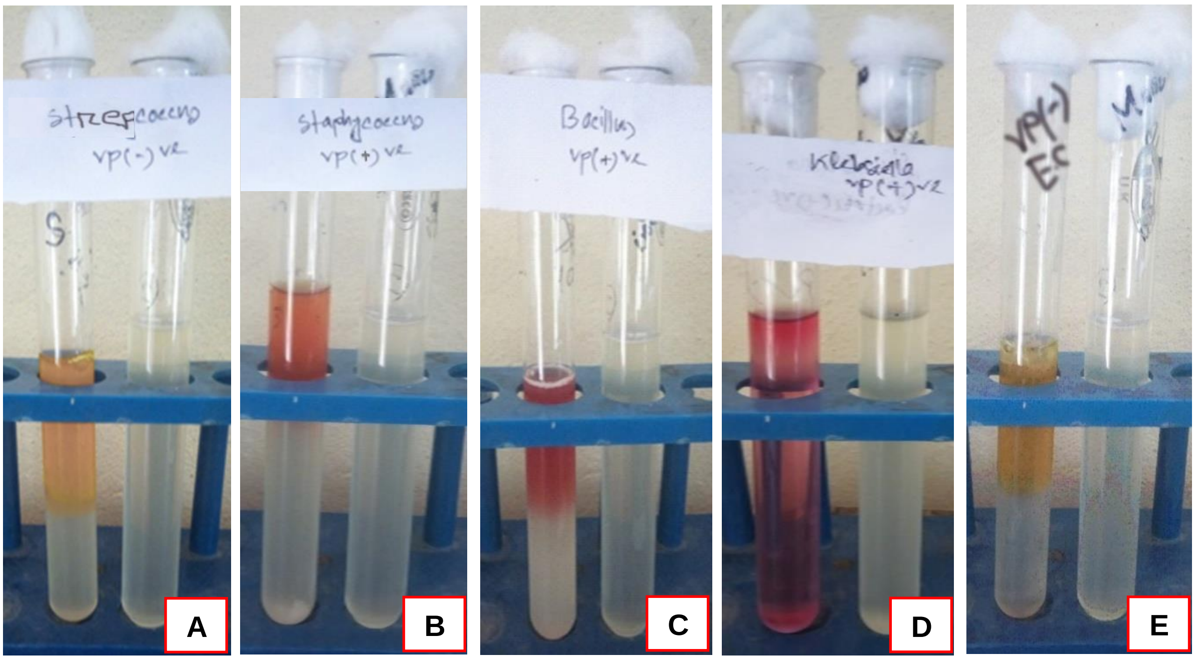

Figure S12: Voges-Proskauer test for biochemical properties of isolated bacteria. A= *Streptococcus spp.*, B= *Staphylococcus spp.*, C= *Bacillus spp.*, D= *Klebsiella spp.*, and E= *E. coli*. Tube B, C and D were positive results indicated red coloration.

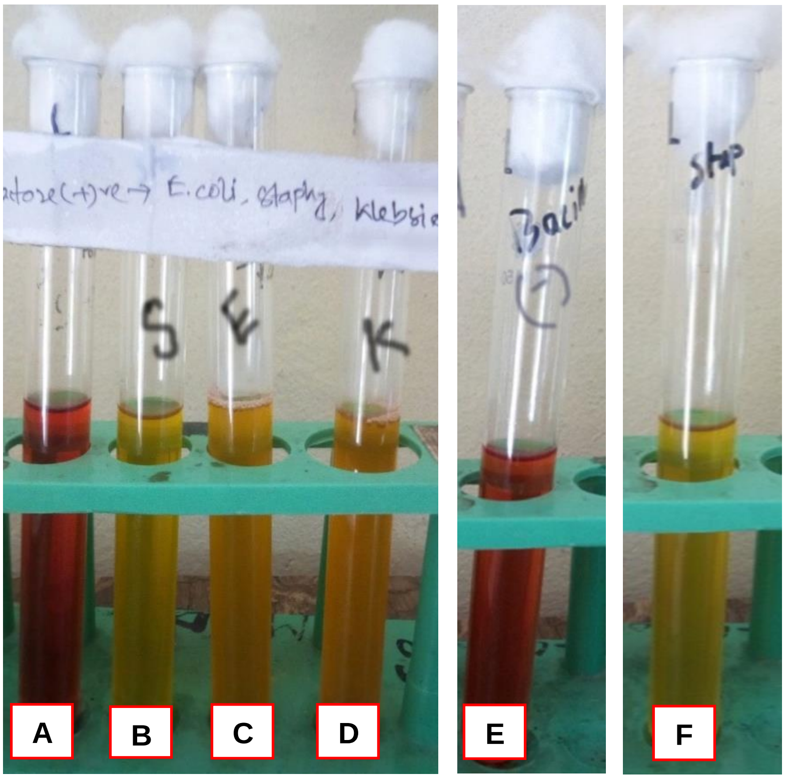

Figure S13: Starch test for biochemical properties of isolated bacteria; A= Control, B= *Staphylococcus spp*., C= *E. coli*, D= *Klebsiella spp*., E= *Bacillus spp*., and F= *Streptococcus spp*. *Bacillus spp.* (tube E) don’t farment suger that indicated positive results.
